# Shielding Human Adipocytes From Inflammation: The Protective Potential of Polyphenol‐Rich *Opuntia ficus‐indica* Cladode Extract

**DOI:** 10.1002/mnfr.70114

**Published:** 2025-05-16

**Authors:** Stefano Quarta, Nadia Calabriso, Maria Annunziata Carluccio, Clara Albano, Ibrahim Khalifa, Martin Wabitsch, Federica Blando, Marika Massaro

**Affiliations:** ^1^ National Research Council (CNR) Institute of Clinical Physiology Lecce Italy; ^2^ National Research Council (CNR) Institute of Sciences of Food Production (ISPA) CNR Lecce Italy; ^3^ Food Technology Department Faculty of Agriculture Benha University Moshtohor Egypt; ^4^ Division of Pediatric Endocrinology Diabetes and Obesity Department of Pediatrics and Adolescent Medicine University of Ulm Ulm Germany

**Keywords:** adipocytes, eucomic acids, inflammation, isorhamnetin, *Opuntia ficus‐indica* (L.) Mill, piscidic

## Abstract

*Opuntia ficus‐indica* (OFI) has attracted much attention as a source of antioxidant and antiinflammatory compounds. We hypothesize that the antioxidant content of OFI cladode extract may improve adipocyte dysfunction resulting from inflammatory stimulation of hypertrophic adipocytes. To this end, the properties of OFI cladode hydroalcoholic extract were evaluated in terms of antioxidant activity, regulation of adipocyte inflammation, and adipocyte/monocyte interaction in human adipocytes rendered dysfunctional by the proinflammatory cytokine tumor necrosis factor‐α (TNF‐α). The major phenolic compounds identified were isorhamnetin derivatives and phenolic acids, including piscidic and eucomic acids. Our results show that OFI cladode extract exhibits antiradical activities and reduces the adhesion and transmigration activity of monocytes to inflamed adipocytes by inhibiting various cytokines, chemokines, and adhesion molecules such as interleukin (IL)‐6 and IL‐8 by ∼80%, monocyte chemotactic protein (MCP)‐1, C‐X‐C motif chemokine ligand (CXC‐L)10, macrophage colony‐stimulating factor (M‐CSF) from 40% to 50%, and intercellular adhesion molecule‐1 (ICAM‐1) by 70% at the higher concentration. In structurally and mechanistically by protein–ligand docking profiling study, piscidic acid proved to be the best potential candidate for a regulatory interaction with the activities of nuclear factor erythroid 2‐related factor 2 (NRF‐2) and nuclear factor‐κB (NF‐κB). In summary, these data highlight the potential of OFI as a dietary supplement in nutritional treatments aimed at combating the inflammatory stigmata of obesity.

## Graphical abstract

Inflammation of the adipose tissue contributes to metabolic disorders. OFI‐CE regulates the expression of proinflammatory genes in human hypertrophic fat cells. OFI‐CE is a promising candidate for use as a dietary supplement or therapeutic agent in nutritional interventions targeting inflammatory markers associated with obesity. DW, dry weight; IL, interleukin; OFI‐CE, *Opuntia ficus‐indica* cladode extracts; ORAC, oxygen radical absorbance capacity; TAEC, trolox‐equivalent antioxidant capacity; TNF‐α, tumor necrosis factor‐α; TPC, total phenol content.

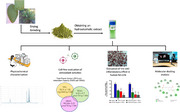

**Abbreviations**
CSF1/M‐CSF: colony‐stimulating factor 1/macrophage colony‐stimulating factor
CXC‐L10: C‐X‐C motif chemokine ligand 10
ICAM‐1: intercellular adhesion molecule‐1
IL‐6: interleukin‐6
IL‐8: interleukin‐8
MCP‐1/CCL‐2: monocyte chemoattractant protein‐1/C‐C motif chemokine ligand 2
NF‐κB: nuclear factor‐κB
NRF‐2
: nuclear factor erythroid 2‐related factor 2
OFI: *Opuntia ficus‐indica* (L.) Mill
SGBS: Simpson–Golabi–Behmel syndrome
TNF‐α: tumor necrosis factor‐α

## 1 Introduction

Obesity is now widely recognized as a growing global public health concern affecting both developed and developing countries [1]. According to recent data from the World Health Organization (WHO), the prevalence of obesity has significantly and consistently increased, tripling over the past two decades [2].

Obesity is a multifaceted and complex disease, influenced by both genetic predispositions and environmental factors, which together contribute to its manifestation [3]. It is triggered by a chronic imbalance between caloric intake and energy expenditure, often associated to the consumption of low‐quality foods [4]. Pathogenically, it is characterized by adipocyte hypertrophy associated with systemic and locally dysregulated production of inflammatory mediators and adipokines [5]. From a mechanistic perspective, dysfunctional adipose tissue (AT) remodeling during obesity activates multiple intrinsic and extrinsic signaling pathways, including the nucleotide‐binding domain and leucine‐rich repeat‐containing protein 3 (NLRP3) and various mitogen‐activated protein kinase (MAPK) pathways. These “molecular sensors/transducers” are capable of recognize perturbed cellular conditions, including endoplasmic reticulum (ER) stress [6] and cause deregulation of proinflammatory and metabolic transcription factors such as nuclear factor‐κB (NF‐κB), activator protein 1 (AP‐1), peroxisome proliferator‐activated receptor (PPAR)γ, and nuclear factor erythroid 2‐related factor 2 (NRF‐2), the latter of which is associated with suppression of proinflammatory NF‐κB activity [7, 8, 9]. The alteration of cellular redox balance plays a crucial role in obesity‐induced inflammation. Obesity‐driven oxidative stress in AT is considered a key factor contributing to cellular dysfunction, insulin resistance, and diabetes [10]. Exposure of cultured adipocytes to H_2_O_2_ impairs insulin‐induced activation of glucose transporter type 4 (GLUT4) [11]. In addition, studies have shown that ER stress and inflammatory responses occur in AT of mice fed a high‐fat diet, likely triggered by free fatty acid (FFA) [12] or tumor necrosis factor‐α (TNF‐α) [13] mediated overproduction of reactive oxygen species (ROS). Increased ROS levels in AT activate NF‐κB and MAPKs, leading to the downregulation of antiinflammatory adipokines and the upregulation of proinflammatory cytokines and chemotactic mediators [14], which promote monocyte infiltration into AT and their differentiation into proinflammatory M1 macrophages [15]. This process has been demonstrated in both animal models of obesity and human studies [16]. Infiltrating immune cells further contribute not only to cytokine production but also to actively release metalloproteinases (MMPs), ROS, and chemokines involved in tissue remodeling, cell signaling, and metabolic regulation of AT [17]. The infiltration of inflammatory cells into AT affects neighboring tissues and organs. In the blood vessels, inflammation of perivascular AT leads to vascular remodeling, increased superoxide production, and endothelial dysfunction, resulting in reduced nitric oxide (NO) availability [18]. This process significantly contributes to the development of vascular diseases, including atherosclerosis and the formation of unstable plaques [18]. These findings underscore the importance of combating and alleviating the hypertrophic, redox‐regulated inflammatory characteristics of expanded AT [18].

Over the past three decades, weight loss and improvement of metabolic risk factors have been a cornerstone in the treatment of obesity‐related diseases [19]. Achieving weight loss typically involves increased physical activity, reduced food consumption, and modifications in lifestyle behavior [20]. However, these conventional interventions often fall short in maintaining long‐term weight loss for overweight and obese individuals. In fact, a significant majority tends to regain their initial weight within 5 years [21]. As a result, newer modern approaches to obesity treatment have evolved to prioritize research into long‐term, safe, and effective natural products that not only support sustainable weight loss but also improve the management of proinflammatory aspects [22].

*Opuntia ficus‐indica* (OFI) is a globally distributed succulent plant renowned for its versatility in dryland environments. With the global population expanding and increasing pressures on water and soil resources, the significance of OFI's attributes is expected to grow, offering sustainable solutions for diverse agricultural needs [23]. Furthermore, its various parts, such as fruits, seeds, and cladodes, offer a wealth of health benefits that reveal its potential as a functional food or dietary supplement [24]. In particular, OFI cladodes are a rich source of polyphenols, polysaccharides, and soluble dietary fibers, which have been associated with weight control [25]. The high content of antioxidants could help explain the observed protective effects in the treatment of chronic diseases [26], including antiadipogenic effects [27]. However, limited information is currently available regarding the antiinflammatory potential of OFI in obesity, especially at the molecular and biochemical levels.

We hypothesize that the antioxidant content of OFI extract may also alleviate the inflammatory stigmata that accompanies hypertrophic adipocytes. To this end, we investigated whether and how OFI cladode extract exhibits antiinflammatory properties in inflamed hypertrophic human adipocytes.

## 2 Materials and Methods

### 2.1 Materials

Human insulin was obtained from Roche Diagnostics (Mannheim, Germany). TNF‐α was purchased from Sigma–Aldrich (now Merck, Darmstadt, Germany). Chemicals and reagents used for RNA isolation and real‐time quantitative polymerase chain reaction were obtained from Bio‐Rad Laboratory (Hercules, CA, USA). Enzyme‐linked immunosorbent assay (ELISA) kits were purchased from Boster Bio (Pleasanton, CA, USA). Unless otherwise indicated, all other reagents were purchased from Sigma–Aldrich.

### 2.2 *Opuntia ficus‐indica* Cladode Extract (OFI‐CE) Preparation

OFI cladodes at the immature stage (fully developed cladodes at 3–4 weeks of development) were collected in spring 2023 from plants (orange variety) grown in the Salento University Botanical Garden (Lecce, Italy). Plant material preparation and polyphenol extraction were done as previously reported [28]. Briefly, the extraction was done in triplicate from 100 mg (DW) macerated with 5 mL aqueous methanol (80%) overnight at 4°C. After centrifugation (4000 × *g*), the supernatant was recovered and evaporated in vacuo at 32°C using an R‐205 Büchi rotavapor (Büchi Labortechnik AG, Essen, Germany). The extract (OFI‐CE) was made sterile by filtering on 0.2 µm‐filter before using it in cell culture experiments.

### 2.3 Cell Culture and Treatment

Simpson–Golabi–Behmel syndrome (SGBS) preadipocytes were cultured and differentiated into mature adipocytes as previously described [29]. To induce inflammation, we stimulated mature adipocytes with TNF‐α, a proinflammatory cytokine whose levels are elevated in the plasma and serum of overweight and obese patients [30]. A 24‐h treatment with 10 ng/mL TNF‐α was chosen on the basis of a pilot dose‐ and time‐course study, showing maximal induction of both monocyte chemoattractant protein‐1 (MCP‐1) protein and mRNA levels after 10 ng/mL TNF‐α for 18 h, in the absence of any effect on cell viability (data not shown). Unstimulated controls were adipocytes incubated without TNF‐α. For OFI‐CE treatment, fully differentiated SGBS cells were incubated with 50–200 µg/mL OFI‐CE for 4 h before stimulation with TNF‐α. THP‐1 cells, as a model of human monocytic cells, were purchased from the American Tissue Culture Collection (Rockville, MD, USA) and cultured in RPMI 1640 as previously described [29].

### 2.4 Cell Viability

The cell viability was determined by a 3‐(4,5‐dimethylthiazol‐2‐yl)‐2,5‐diphenyl tetrazolium bromide (MTT) assay, based on the ability of viable cells to convert MTT, a soluble tetrazolium salt, into an insoluble formazan precipitate, which is then quantified spectrophotometrically. After OFI‐CE treatment and TNF‐α stimulation, mature adipocytes were incubated with MTT (0.5 mg/mL) for 2 h, and the formazan products were then dissolved in isopropanol. The absorbance was measured at 595 nm by a microplate reader.

### 2.5 Identification and Quantification of Phenolic Compounds

The identification and quantification of phenolic compounds in OFI‐CE were performed using an Agilent 1100 Liquid Chromatography system (Agilent Technologies, Palo Alto, CA, USA). The chromatographic conditions and column were the same reported in De Bellis et al. [31]. Briefly, the mobile phase was (A) H_2_O/formic acid: 95/5 and (B) acetonitrile/formic acid: 95/5. The gradient proceeded as following: 1 min of isocratic elution at 7% B, 25 min of increasing gradient up to 17% B, 9 min up to 56% B, 5 min of isocratic elution at 56% B, 3 min up to 80% B, and 8 min of isocratic elution at 80% B, with a flow rate 1 mL/min. Before the next injection an equilibration time of 10 min occurred. The column was a C18 Luna (Phenomenex, 250 Å ∼ 46 mm, 5 µm) in conjunction with a C18 guard cartridge column, both thermostated at 30°C.

The polyphenolic compounds were identified by comparing their peak retention times and UV–visible spectra with those of commercial standards, where available. For piscidic and eucomic acids, the *p*‐hydroxybenzoic acid standard curve (*y* = 44 661*x* − 16.2; *r* ^2^ = 0.9997, six points) was used, as they share a similar chemical structure and spectrum. For isorhamnetin derivatives, the isorhamnetin 3‐rutinoside standard curve (*y* = 41 751*x* + 63.5; *r* ^2^ = 0.9999, six points) was used for quantification.

### 2.6 Phenols and Antioxidant Capacity

OFI‐CE was assessed for total phenol content and reducing capacity by the Folin–Ciocalteu (F‐C) assay, as well as their antioxidant capacity using the ABTS assay (trolox‐equivalent antioxidant capacity [TEAC]) and the oxygen radical absorbance capacity (ORAC) assay, as reported [31]. A rapid microplate methodology, using a microplate reader (Infinite M‐200, Tecan Group Ltd, Männedorf, Switzerland) and 96‐well plates (Costar, 96‐well clear round bottom plate, for Folin and ABTS, black round bottom plate, for ORAC), was used. Two independent plates were prepared, testing OFI‐CE in triplicate with three dilutions.

### 2.7 RNA Isolation and Real‐Time Quantitative Polymerase Chain Reaction

The total RNA was isolated by using PureZol RNA isolation reagent (Bio‐Rad Laboratory), according to the manufacturer's protocol. One microgram of RNA was used for the cDNA synthesis using iScript cDNA Synthesis Kit (Bio‐Rad Laboratory). The reaction was carried out on a GeneAmp PCR System 9700 (Applied Biosystems) under the following conditions: 5 min at 25°C, 20 min at 46°C, and 1 min at 95°C. Quantitative real‐time PCR (qPCR) analyses were performed with the CFX96 Touch Real‐Time PCR Detection System instrument and software (Bio‐Rad Laboratory). All the reactions were performed in a total volume of 25 µL containing 50 ng of cDNA, 0.3 pmol/L of a primer pair, and 12.5 µL of the 2× Sso Advanced Universal SYBR Green Supermix (Bio‐Rad Laboratory) mix under the following conditions: 2 min at 50°C, 10 min at 95°C, and 40 cycles of 15 s at 95°C and 1 min at 60°C. The reactions were carried out in triplicate on three independent sets of RNA. Negative controls (no RNA added) were processed under the same conditions as the experimental samples. The amount of mRNA was calculated by using the comparative critical threshold (CT) method. To account for possible variations related to cDNA input or the presence of PCR inhibitors, the endogenous reference gene ribosomal 18S was simultaneously quantified for each sample, and the data were normalized accordingly. The primer sequences used are shown in Table 1.

**TABLE 1:** Primer sequences used for qPCR analysis.

Gene symbol	Full name	Forward primer	Revers primer	Accession number
IL‐6	Interleukin‐6	AGGAGACTTGCCTGGTGAAA	CAGGGGTGGTTATTGCATCT	NM_000600.5
IL‐8	Interleukin‐8	GTGCAGTTTTGCCAAGGAGT	CTCTGCACCCAGTTTTCCTT	NM_001354840.3
MCP‐1/CCL‐2	Monocyte chemoattractant protein‐1/C‐C Motif chemokine ligand 2	CCCCAGTCACCTGCTGTTAT	TCCTGAACCCACTTCTGCTT	NM_002982.3
CXC‐L10	C‐X‐C motif chemokine ligand 10	CAAGGATGGACCACACAGAG	GCAGGGTCAGAACATCCACT	NM_001565.2
CSF1/M‐CSF	Colony‐stimulating factor 1/macrophage colony‐stimulating factor	TGGACGCACAGAACAGTCTC	CCTCCAGGGCTCACAATAAA	NM_000757.4
ICAM‐1	Intercellular adhesion molecule‐1	AGACATAGCCCCACCATGAG	CAAGGGTTGGGGTCAGTAGA	NM_000201.2
18S	18 Ribosomal RNA	AAACGGCTACCACATCCAAG	CCTCCAATGGATCCTCGTTA	NR_003286.2

### 2.8 Measurement of MCP‐1 Protein Release

Conditioned medium from fully differentiated adipocytes treated with OFI‐CE and then stimulated with TNF‐α was collected, and the levels of secreted MCP‐1 were determined using the corresponding ELISA kit (Boster Bio), according to the manufacturer's instructions (catalog number EK0441).

### 2.9 THP‐1 Adhesion Assay

THP‐1 cells (10^6^ cells/mL) were fluorescently labeled by incubation with calcein‐AM (5 ng/mL) for 30 min and then washed twice in RPMI medium. As previously described [29], suspended THP‐1 cells were then added to the SGBS monolayers treated with OFI‐CE and then stimulated with TNF‐α for 1 h. The nonadhering monocytes were removed by gentle washing with DMEM‐F12 and monolayers were fixed with 1% paraformaldehyde. Images of SGBS and adherent calcein‐labeled THP‐1 cells were visualized and captured with a stereomicroscope (Nikon, Minato, Tokyo, Japan), equipped with Nikon NIS‐Elements D at 40× magnification. Finally, adherent monocytes were counted using the ImageJ program (http://imagej.nih.gov/ij/, accessed on July 23, 2023).

### 2.10 In Vitro THP‐1 Chemotaxis Assay

For the preparation of adipocytes‐conditioned medium, differentiated SGBSs were treated with OFI‐CE before TNF‐α stimulation. Media were collected under sterile conditions, centrifuged to remove cell debris, and frozen at −20°C until the chemotaxis assay was performed. Adipocyte medium was placed in the lower chamber of a transwell system (Corning purchased through Sigma–Aldrich, St. Louis, MO, USA), while the monocytes were placed in the upper chamber. After 1 h, the monocytes that had migrated to the lower chamber were measured as previously described [32].

### 2.11 In Silico Molecular Docking

Molecular docking is a computational modeling technique that allows the prediction of molecular interactions that hold together a protein and a ligand in the bound state [33]. The crystal structures of the selected target proteins were derived from the Protein Data Bank (PDB, www.wwpdb.org) (accessed on September 20, 2024) with PDB IDs as follows: 1FOS for AP‐1, 4Q3J for NF‐κB, and 4L7B for NRF‐2. To explore the potential molecular mechanisms by which OFI‐CE influences adipocyte physiology under proinflammatory conditions, we investigated the ability of its key components to interact with transcription factors involved in inflammation and oxidative stress. Specifically, we examined the interaction of eucomic acid, ferulic acid, and piscidic acid with NF‐κB, NRF‐2, and AP‐1.

In order to prepare the proteins for the docking simulation, all the missing atoms were repaired. In addition, all the water molecules and the cocrystalized heteromolecules were removed, followed by the addition of polar hydrogen atoms and neutralization using Kollman united‐atom charges. The dimensions of the grid box were 60 × 60 × 60 with a 0.500 Å distance between the points. During the docking procedure, the ligand was flexible and the protein was rigid. AutoDock4 and Lamarckian genetic algorithms were used to dock 250 conformations. The best docked pose was saved, and the results of the best poses for the proteins with the ligands were analyzed using the free energy of binding (Δ*G*) and the inhibition constant (*K*_i_). Discovery Studio 2020 Visualizer was used to investigate the protein–ligand nonbonding interactions of the best poses. The docking calculations were conducted using AutoDock Tools 1.5.6.

### 2.12 Statistical Analysis

The results were expressed as means ± SD. Student's *t* test was used to compare the means between the control group and the compound‐treated group. A *p* value of <0.05 was considered statistically significant.

## 3 Results

### 3.1 Identification and Quantification of Phenolic Compounds

**FIGURE 1:**
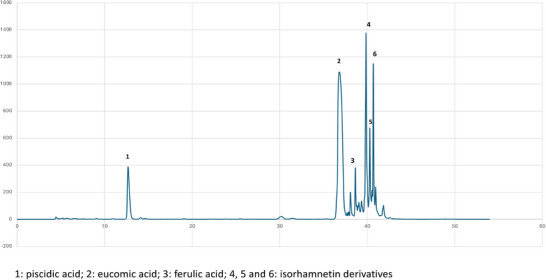
HPLC representative chromatogram of the OFI‐CE detected at *λ* = 280 nm. OFI‐CE, *Opuntia ficus‐indica* (L.) Mill.

**FIGURE 2:**
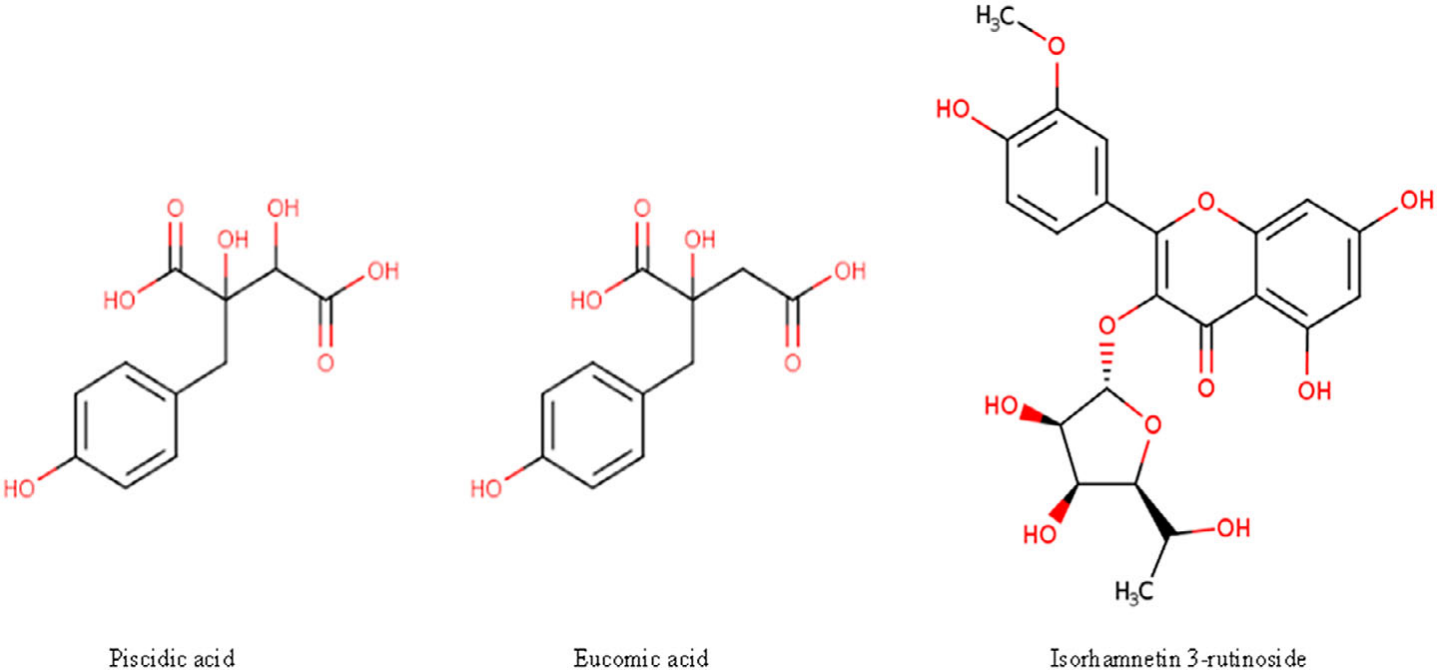
Chemical structure of piscidic and eucomic acids and isorhamnetin 3‐rutinoside.

The hydroalcoholic extract of OFI cladode proved to be rich of polyphenols. We chose the immature stage (3–4 months old pads) as in our previous work, we found this development stage the richest of phenolics [28]. The analysis by HPLC of OFI‐CE revealed the presence of different polyphenolic compounds, which were identified upon their retention time and UV–vis spectra in comparison with authentic standard, or with the available literature [28, 34]. Major peaks were identified corresponding to phenolic acids (piscidic, eucomic, and ferulic acids) and flavonols (isorhamnetin derivatives) (Figures 1 and 2). As reported in M&M, piscidic and eucomic acids were quantified by the *p*‐hydroxybenzoic acid standard curve; for isorhamnetin derivatives, the isorhamnetin 3‐rutinoside spectrum matched the spectra of Peaks 4, 5, and 6; therefore, the isorhamnetin 3‐rutinoside standard curve was used for quantification. Table 2 presents the quantification of the major phenolic compounds identified in OFI‐CE. Notably, eucomic acid was detected in a significant amount, as already reported in our previous study in immature cladode [28], while piscidic acid is usually predominant in mature cladode [28]. Also, isorhamnetin derivative compounds were abundant in OFI‐CE.

**TABLE 2:** Quantification of the major phenolic compounds identified in OFI‐CE and its antioxidant capacity.

Major phenolics amount (mg/g DW)	
Piscidic acid	1.45 ± 0.03
Eucomic acid	8.57 ± 0.33
Isorhamnetin derivatives	11.2 ± 0.52
Total phenol content (TPC) and antioxidant capacity	
TPC	30.31 ± 1.52 mg GAE/g DW
TEAC	6.61 ± 0.7 µmol TE/g DW
ORAC	763.86 ± 5.22 µmol TE/g DW

*Note*: Piscidic and eucomic acids content is expressed as mg *p*‐hydroxybenzoic acid equivalent/g, dry weight (DW) material. Isorhamnetin derivatives content is reported as mg isorhamnetin 3‐rutinoside equivalent. Total phenol content (TPC) as mg gallic acid equivalent (GAE)/g DW. Antioxidant capacities (by TEAC and ORAC assays) are expressed as µmol Trolox equivalent (TE)/g DW. Values represent mean ± SD (*n* = 3).

To evaluate the antioxidant potential of an extract, it has been recommended that applications for three assays (that vary in their mechanisms of antioxidant action) be considered for standardization:  the ORAC assay, the F‐C method, and the TEAC assay [35]. For this reason, we assessed the antioxidant capacity of OFI‐CE using both the TEAC assay (a single electron transfer [ET] reaction‐based assay) and the ORAC assay (a hydrogen atom transfer [HAT] reaction‐based assay). The F‐C assay can also be considered an ET‐assay, as it measures the reducing capacity of the sample [36]. Results are shown in Table 2, revealing a good phenolic content (by Folin assay) and an exceptionally high ORAC value.

### 3.2 Effect of OFI‐CE on Cell Viability

**FIGURE 3:**
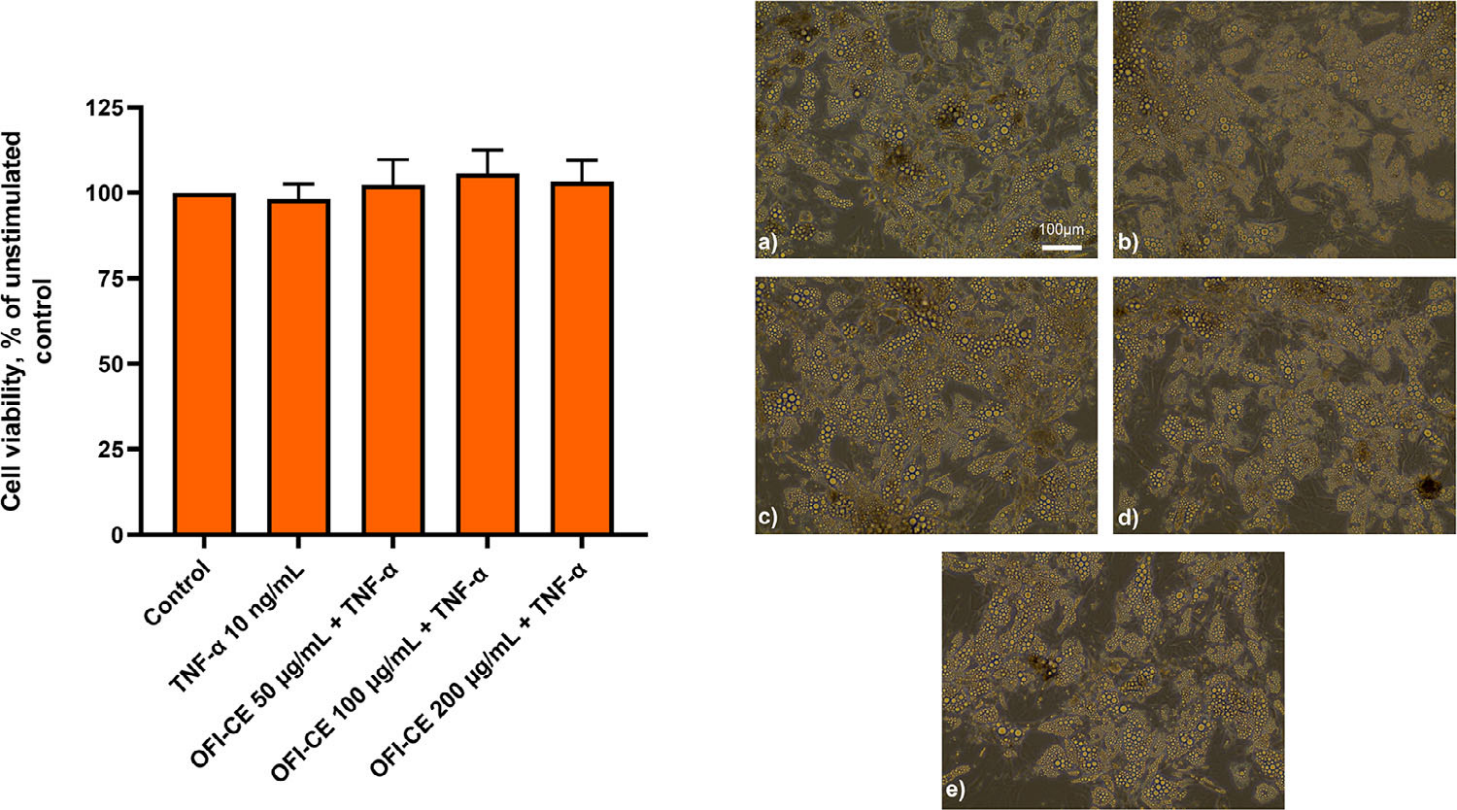
The effect of OFI‐CE treatment on cell viability. SGBS adipocytes were treated with OFI‐CE for 4 h at the concentrations indicated, and then either treated with 10 ng/mL TNF‐α or left untreated for 18 h. Cell viability was assessed by the MTT assay (left panel). Data (means ± SD, *n* = 3) are expressed as a percentage of the unstimulated control. Right panels, SGBS images were visualized and acquired with a phase contrast microscope at 10× magnification. (a) Control, (b) TNF‐α 10 ng/mL, (c) OFI‐CE 50 µg/mL + TNF‐α, (d) OFI‐CE 100 µg/mL + TNF‐α, and (e) OFI‐CE 200 µg/mL + TNF‐α. For statistical analysis, we used Student's *t* test. MTT, 3‐(4,5‐dimethylthiazol‐2‐yl)‐2,5‐diphenyl tetrazolium bromide; OFI‐CE, *Opuntia ficus‐indica* (L.) Mill; SGBS, Simpson–Golabi–Behmel syndrome adipocytes; TNF‐α, tumor necrosis factor‐α.

Preliminary experiments were conducted to evaluate the effects of OFI‐CE on the viability of fully differentiated adipocytes. The extract was tested for cell toxicity at concentrations ranging from 10 to 200 µg/mL in the presence or absence of TNF‐α. After treatment, cell viability was determined using the MTT assay, total protein content, and cell morphology analysis. As shown in Figure 3 (left), OFI‐CE treatment had no effect on cell viability at any of the concentrations tested, either in the absence (data not shown) or presence of TNF‐α. Similar results were observed in morphological examinations (Figure 3, right panels) and at the protein level (data not shown). Therefore, we used OFI‐CE concentrations of 50, 100, and 200 µg/mL in all subsequent experiments.

### 3.3 OFI‐CE Attenuates TNF‐α‐Mediated Inflammatory Gene Expression in Human Adipocytes

**FIGURE 4:**
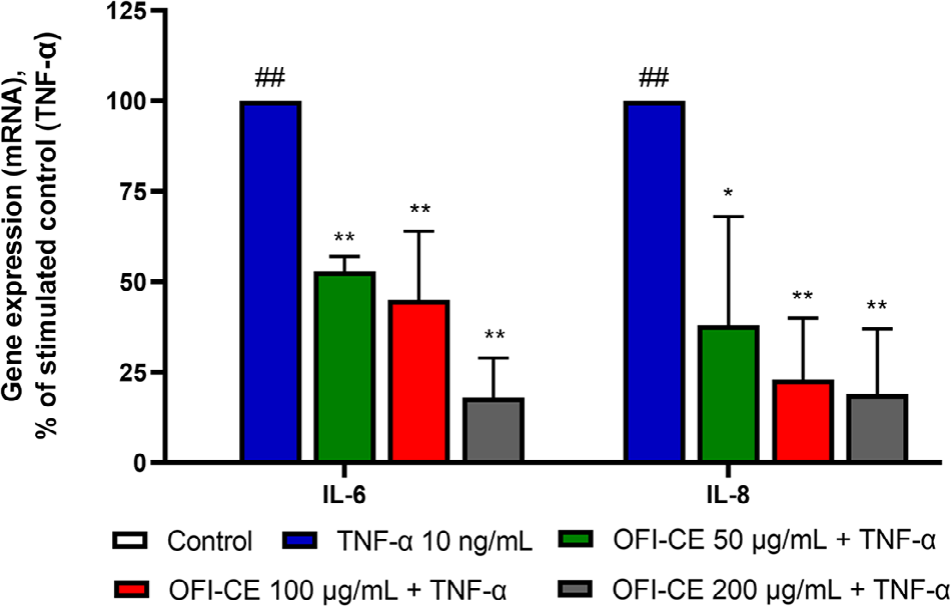
OFI‐CE treatment attenuates TNF‐α‐induced expression of IL‐6 and IL‐8. SGBSs were treated with OFI‐CE at the concentrations indicated for 4 h and then either treated with 10 ng/mL TNF‐α or left untreated for 18 h. Total RNA was extracted from cells, and mRNA levels of IL‐6 and IL‐8 were measured by qPCR using specific primers and normalized to 18S RNA. Data (means ± SD, *n* = 3) are expressed as percentage over TNF‐α alone. ##*p* < 0.01 versus basal (untreated) control; *p < 0.05 versus TNF‐α alone; **p < 0.01 versus TNF‐α alone. For statistical analysis, we used Student's *t* test. IL‐6, interleukin‐6; IL‐8, interleukin‐8; OFI‐CE, *Opuntia ficus‐indica* (L.) Mill; TNF‐α, tumor necrosis factor‐α.

**FIGURE 5:**
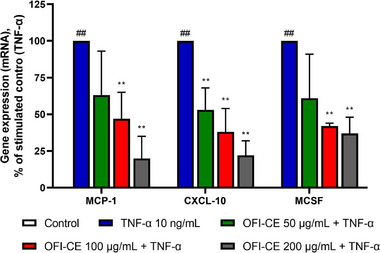
OFI‐CE treatment counteracts TNF‐α‐induced expression of chemoattractant genes. SGBSs were treated with OFI‐CE at the concentrations indicated for 4 h and then either treated with 10 ng/mL TNF‐α or left untreated for 18 h. Total RNA was extracted from cells, and mRNA levels of MCP‐1, CXCL‐10, and M‐CSF were measured by qPCR using specific primers and normalized to 18S RNA. Data (means ± SD, *n* = 3) are expressed as percentage over TNF‐α alone. ##*p* < 0.01 versus basal (untreated) control; **p < 0.01 versus TNF‐α alone. For statistical analysis, we used Student's *t* test. OFI‐CE, CXC‐L10, C‐X‐C motif chemokine ligand 10; MCP‐1, monocyte chemoattractant protein‐1; M‐CSF, macrophage colony‐stimulating factor; *Opuntia ficus‐indica* (L.) Mill; TNF‐α, tumor necrosis factor‐α.

We evaluated the effect of OFI‐CE on TNF‐α‐induced gene expression of a panel of proinflammatory cyto/chemokine (Figures 4 and 5). Interleukin‐6 (IL‐6) and 8 (IL‐8) showed higher expression in the AT of obese subjects and are involved in the recruitment of immune cells and in the pathogenesis of obesity and insulin resistance [37]. TNF‐α induced the expression of both cytokines in human adipocytes. However, pretreatment of cells with OFI‐CE effectively countered the TNF‐α‐induced expression of IL‐6 and IL‐8 in a dose‐dependent manner (Figure 4). TNF‐α, also produced by adipocytes themselves, induces the expression of chemoattractant proteins able to recruit circulating monocytes and immune cells to the AT. As shown in Figure 5, exposure to OFI‐CE attenuates, in a dose‐dependent manner, the TNF‐α induced expression of MCP‐1, CXC‐L10, and macrophage colony‐stimulating factor (M‐CSF).

### 3.4 OFI‐CE Attenuates Chemiotaxis and Monocyte Adhesion to Inflamed Adipocytes

**FIGURE 6:**
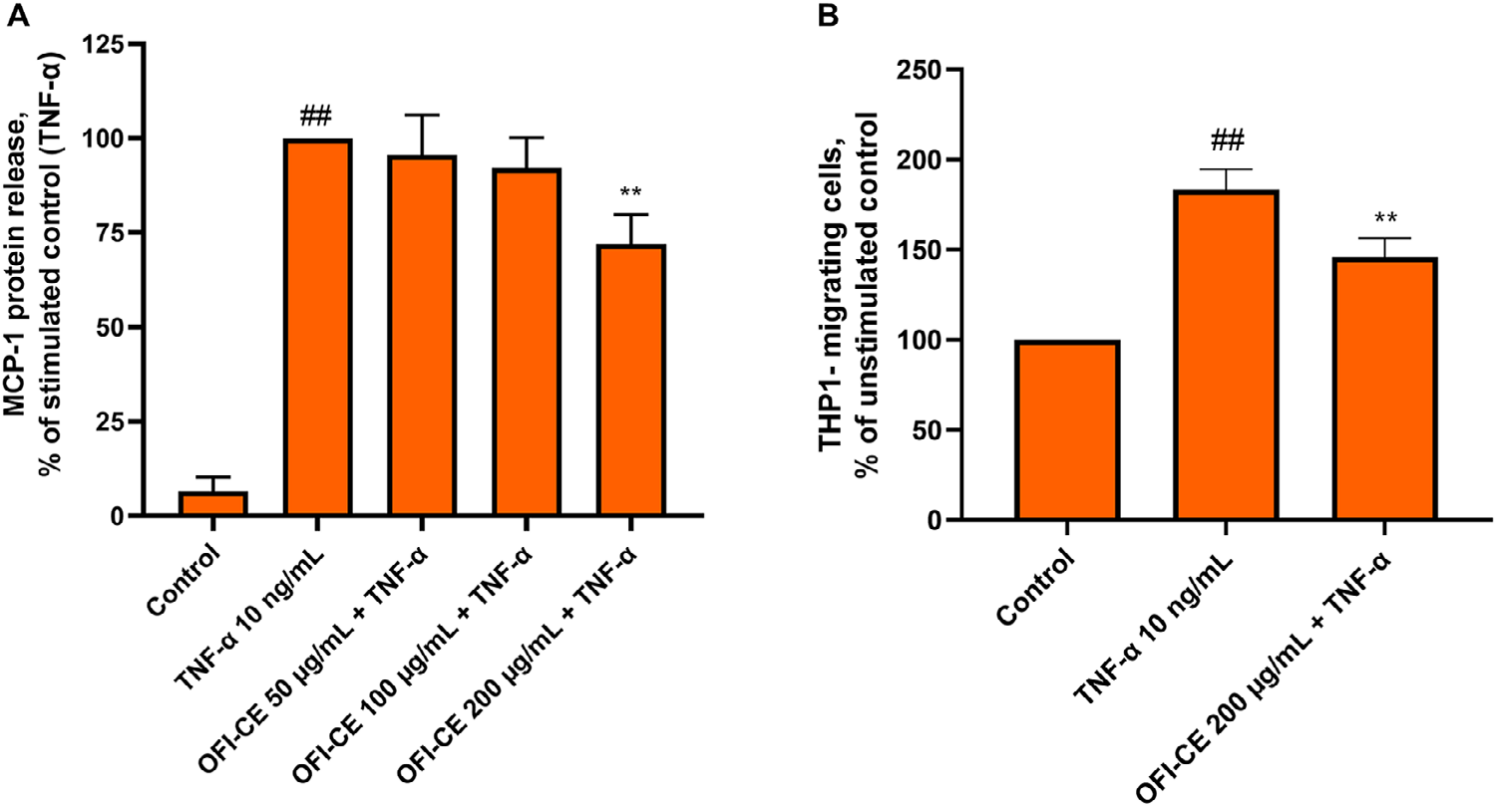
OFI‐CE treatment reduced TNF‐α‐induced release of MCP‐1 and chemiotaxis. SGBSs were treated with OFI‐CE at the concentrations indicated for 4 h and then either treated with 10 ng/mL TNF‐α or left untreated for 18 h. (A) Culture medium was collected, and MCP‐1 release was evaluated by ELISA assay. Data (mean ± SD, *n* = 3) are expressed as a percentage over TNF‐α alone. ##*p* < 0.01 versus basal (untreated) control; **p < 0.01 versus TNF‐α alone. (B) Culture medium was collected and added to the lower chamber in a Boyden chamber. THP‐1 (2.5 × 10^6^ cells/mL) were added to the upper chamber. After 60 min, migrated THP‐1 cells were measured by MTT assay. Data (means ± SD, *n* = 3) are expressed as the number of migrated monocytes over TNF‐α alone. ##*p* < 0.01 versus basal (untreated) control; **p < 0.05 versus TNF‐α alone. For statistical analysis, we used Student's *t* test. MCP‐1, monocyte chemoattractant protein‐1; MTT, 3‐(4,5‐dimethylthiazol‐2‐yl)‐2,5‐diphenyl tetrazolium bromide; OFI‐CE, *Opuntia ficus‐indica* (L.) Mill; TNF‐α, tumor necrosis factor‐α.

**FIGURE 7:**
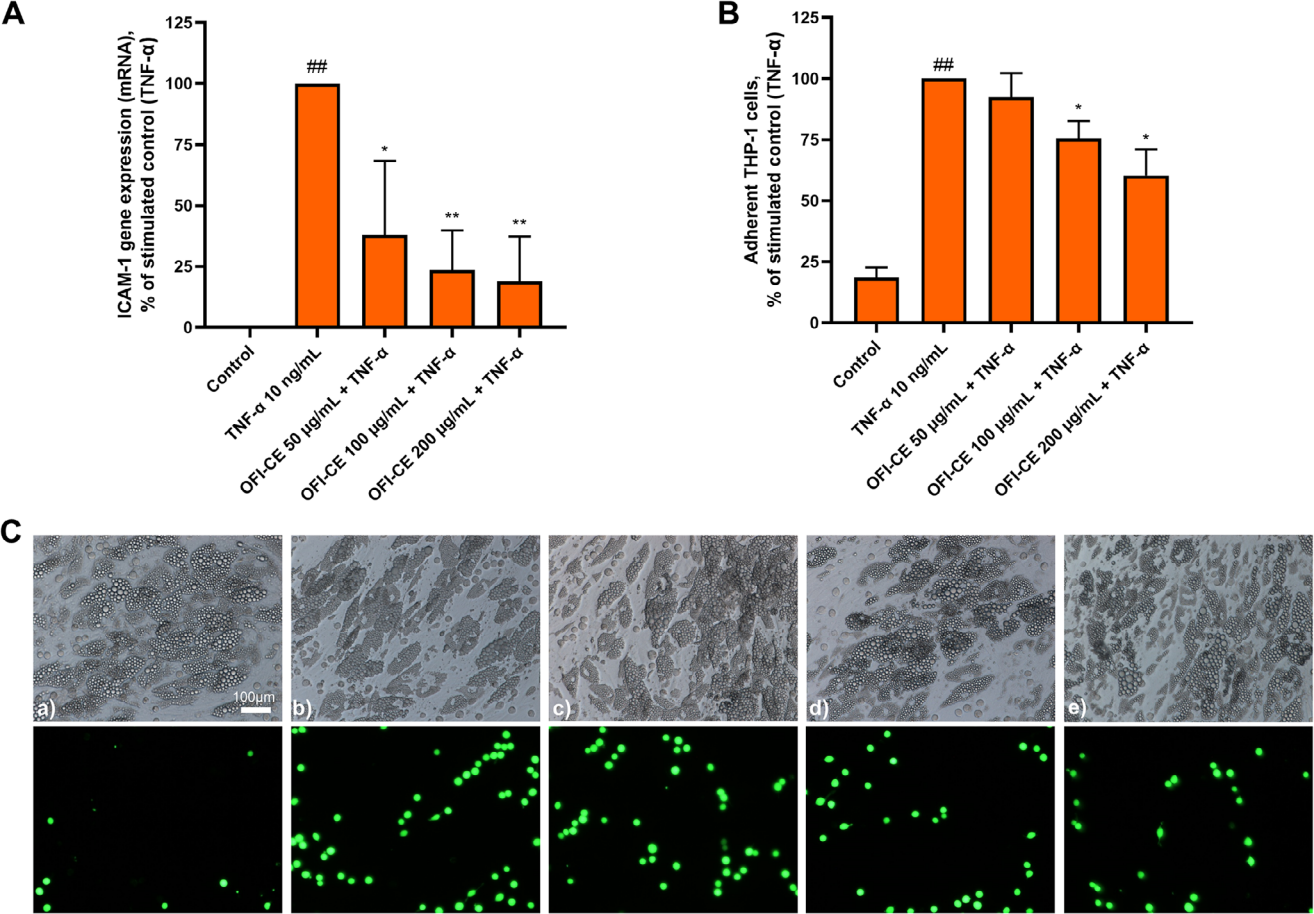
OFI‐CE counteracts TNF‐α‐induced ICAM‐1 expression and monocytes adhesion to inflamed adipocytes. SGBSs were treated with OFI‐CE at the concentrations indicated for 4 h and then either treated with 10 ng/mL TNF‐α or left untreated for 18 h. (A) Total RNA was extracted from cells, and mRNA levels of ICAM‐1 were measured by qPCR using specific primers and normalized to 18S RNA. Data (means ± SD, *n* = 3) are expressed as percentage over TNF‐α alone. ##*p* < 0.01 versus basal (untreated) control; *p < 0.05 versus TNF‐α alone; **p < 0.01 versus TNF‐α alone. (B, C) Fluorescently labeled THP‐1 (106 cells/mL) was added to the SGBS monolayers. After 1 h, nonadhering cells were removed by three washes, and images of SGBS and adherent calcein‐labeled THP‐1 cells were visualized and captured with a fluorescent microscope at 10× magnification. (a) Control, (b) TNF‐α 10 ng/mL, (c) OFI‐CE 50 µg/mL + TNF‐α, (d) OFI‐CE 100 µg/mL + TNF‐α, and (e) OFI‐CE 200 µg/mL + TNF‐α. Data (means ± SD, *n* = 3) are expressed as the number of adherent monocytes over TNF‐α alone. ##*p* < 0.01 versus basal (untreated) control; *p < 0.05 versus TNF‐α alone. For statistical analysis, we used Student's *t* test. ICAM‐1, Intercellular adhesion molecule‐1; MTT, 3‐(4,5‐dimethylthiazol‐2‐yl)‐2,5‐diphenyl tetrazolium bromide; OFI‐CE, *Opuntia ficus‐indica* (L.) Mill; TNF‐α, tumor necrosis factor‐α.

To further investigate the antiinflammatory properties of OFI‐CE on inflamed adipocytes, the release of MCP‐1 was measured by ELISA assay. As expected, TNF‐α stimulation induced the expression and release of the chemokine, while cell exposure to the higher OFI‐CE concentration significantly reduces, by ∼30% (*p* <0.01), the secretion of MCP‐1 in the culture media (Figure 6A). As a functional consequence of the ability of OFI‐CE to attenuate the expression of inflammatory genes in TNF‐α‐stimulated adipocytes, we performed a transwell cell migration assay. As shown in Figure 6B, a significant reduction in the number of migrated monocytes was observed (*p* < 0.01). As shown in Figure 7A, TNF‐α stimulation increased ICAM‐1 expression, consequently enhancing THP‐1 monocyte adhesion to adipocytes (Figure 7B,C). However, pretreatment with OFI‐CE resulted in a significant reduction in ICAM‐1 expression across all tested concentrations in adipocytes (*p* <0.01). This reduction in adhesion molecule expression corresponded to a significant decrease in monocyte adherence to inflamed adipocytes (*p* < 0.05).

### 3.5 Molecular Docking Results

**FIGURE 8:**
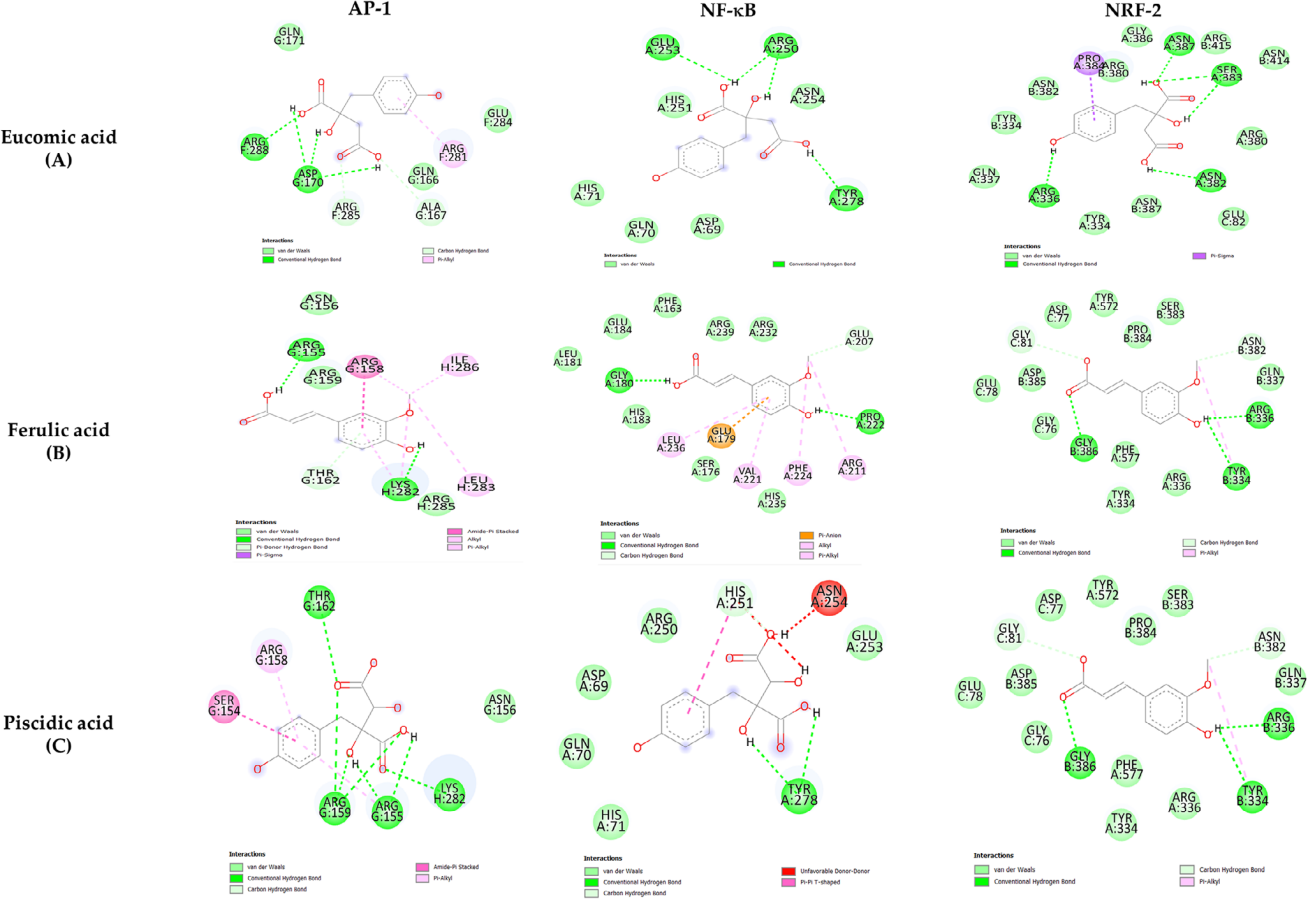
Molecular docking models of putative interactions with target proteins. The binding of eucomic, ferulic, and piscidic acid in the active site of AP‐1 (PDB ID, 1FOS), NF‐κB (PDB ID 4Q3J), and NRF‐2 (PDB ID 4L7B) is reported in 2D conformation. The images were rendered using Discovery Studio. AP‐1, activator protein 1; NF‐κB, nuclear factor‐κB; NRF‐2, nuclear factor erythroid 2‐related factor 2.

Binding affinity, assessed by Δ*G* and *K*_i_ values and covalent, noncovalent, and hydrophobic interactions, was computationally predicted. The visualized 3D molecular docking interaction is shown in Figure 8, and the corresponding affinity/binding values between the investigated compounds and potential ligand molecules are listed in Table 3. The virtual screening of ligand binding activity showed that all tested ligands can interact with the target proteins through both covalent and noncovalent interactions.

**TABLE 3:** AutoDock4 docking results: binding energy and inhibition constant.

	AP‐1		NF‐κB		NRF‐2	
	Binding energy (kcal/mol)	Inhibition constant (*K*_i_)	Binding energy (kcal/mol)	Inhibition constant (*K*_i_)	Binding energy (kcal/mol)	Inhibition constant (*K*_i_)
Eucomic acid	−5.30	130 µM	−6.91	8.66 µM	−7.90	1.62 µM
Ferulic acid	−5.77	59.35 µM	−7.04	6.88 µM	−7.29	4.51 µM
Piscidic acid	−5.99	40.73 µM	−6.67	12.94 µM	−9.46	115.50 nM

Among the target proteins, NRF‐2 showed the strongest interactions with the ligands. In particular, piscidic acid showed a high affinity for NRF‐2 with a *K*_i_ value of 115 nM. For NF‐κB, *K*_i_ values were in the range of 10 µM, indicating that these ligands can bind to the active site of the protein. The weakest affinities were found for AP‐1.

## 4 Discussion

Overall, our data confirm the initial experimental hypothesis that OFI‐CE, with its high antioxidant content, can attenuate the proinflammatory markers characteristic of hypertrophic adipocytes. Within the complex cellular environment of AT, adipocytes and macrophages play a key role in orchestrating the inflammatory dysmetabolism associated with obesity and insulin resistance [38]. In our research, we used the extensively validated fat cell model represented by the human SGBS cells and the monocytic THP‐1 cell line. Upon differentiation, SGBS cells mirror the pathophysiological characteristics of visceral AT and closely replicate the proinflammatory phenotypes observed in abdominal fat accumulation, making them a valuable tool for studying the antiadipogenic and antiinflammatory effects of drugs and bioactive nutrients [39, 40, 41].

Our results suggest that crude extract of OFI cladodes effectively counteracts the proinflammatory activation of fat cells induced by cell exposure to TNF‐α, a cytokine long known to play a role in glycemic control and insulin resistance in obesity [42, 43], but which also responds to dietary intervention [30]. The data collected are consistent with the findings of Matias et al. [44] and Cho et al. [45] who reported a reduction in the expression of the cytokines TNF‐α, IL‐6, and IL‐1β in human colon cancer cells and macrophages, respectively, after exposure to OFI extract. They attributed this effect to a mechanism involving reduced activation of the proinflammatory, redox‐sensitive transcription factor NF‐κB.[44, 45] Consistent with this, our molecular docking prediction analysis confirms the ability of the OFI extract to bind NF‐κB, while suggesting an even stronger affinity for the redox‐sensitive transcription factor NRF‐2. The potential of OFI extract to modulate NRF‐2 activity is consistent with previous observations in mouse models of atherosclerosis that emphasized the role of NRF‐2 in mediating the antiinflammatory and antiatherosclerotic effects of OFI extract [46]. These findings provide the basis for future studies to directly investigate the mechanistic effects of OFI extracts on these transcription factors.

As far as we know, our experiments are the first to investigate and demonstrate an antiinflammatory effect of OFI cladode extract in inflamed human adipocytes. This observation complements previous research that had emphasized an antiadipogenic effect of OFI fruit and cladode extract, as observed by Eseberri et al. [47] and Héliès‐Toussaint et al. [48] in mouse models of adipocytes. Therefore, OFI extracts have the potential as dietary supplements to address the dual challenge of obesity: the accumulation of fat and its associated clinical manifestations, including systemic inflammation and the progression of insulin resistance and atherosclerosis in the arteries, which are precursors to myocardial infarction and stroke. Consistently, in vivo studies, albeit limited in number, have successfully investigated the health‐promoting antiobesity and antiinflammatory properties of phenolic compounds found in OFI, such as piscidic and eucomic acids and isorhamnetin derivatives. Both Zhang [49] and Rodriguez‐Rodriguez [50] have shown that isorhamnetin supplementation on a high‐fat diet helps to prevent weight gain, reduce adipocyte size, and improve lipid and glycemic profile in both female and male rats. Recent studies by Di Majo et al. have confirmed similar findings, demonstrating that, under comparable experimental conditions, in addition to improvements in weight gain, insulin sensitivity, and antioxidant status, there were also notable enhancements in the cognitive functions of rats [51]. Finally, in the rat model of carrageenan‐induced air pouch inflammation, both OFI extracts and extracts enriched with isorhamnetin glycosides were able to reduce local and systemic inflammation [52]. Furthermore, OFI was found to reduce the development of atherosclerotic plaques in apoE knock‐out mice via a mechanism involving reduced activation of endothelial cells, as evidenced by the downregulation of the expression of the adhesion molecules ICAM‐1 and VCAM‐1 [53]. In agreement with Garoby‐Salom et al. [53], we also observe a decrease in adipocyte expression of ICAM‐1, which is functionally associated with a decrease in monocyte adhesion and transmigration. Since OFI extracts have demonstrated efficacy at concentrations achievable in the plasma of healthy individuals [54], our findings suggest that OFI may have real antiinflammatory and vasculoprotective effects.

When performing cell‐based assays to characterize a plant extract, a comprehensive understanding of its composition in terms of bioactive compounds is imperative for a thorough evaluation. Our HPLC analysis of OFI‐CE aligned with the findings of our prior study [28], indicating a significant presence of eucomic acid in the immature cladode extract, along with notable levels of isorhamnetin derivatives all endowed with antioxidant activity that may help explaining the observed biological activity [44]. In this regard, the antioxidant assays we performed to characterize OFI‐CE provide an interesting overview of the biological activities of OFI‐CE. The antioxidant capacity measured with the ABTS method was not very high compared to the total (poly)phenol content, as it is well know the correlation between antioxidant capacity and total phenol content (assayed by F‐C method). In contrast, the antioxidant capacity measured with the ORAC assay was exceptionally high and in line with previously reported values [55]. This could be due to the mechanism of hydrogen donation, which is able to quench free radicals, in relation to the specific structure of the major phenolic acids in OFI‐CE (piscidic and eucomic acids) which promote the HAT to free radical [55] and potentially reduce the activation of redox‐sensitive signaling pathways/transcription factors involved in the activation of proinflammatory gene expression [56]. The ORAC assay represents a biologically relevant test, which measures the antioxidant inhibition of peroxyl radical‐induced oxidations by HAT [35]. The accountability of the ORAC assay relies on the evaluation of probe reaction with peroxyl radicals (a physiological radical source) over time through the loss of fluorescence. Moreover, the reaction buffer is heated to a physiological temperature of 37°C. Therefore, the assay accounts for lag time, initial rate, and total extent of inhibition in a single value, in a biologically relevant environment [57]. In this sense, the low antioxidant capacity observed in the ABTS assay could be due to the mechanism of the assay itself. The reaction involving the radical cation (ABTS•+) may not proceed at the same rate for slower reactions, and it might take a longer time to reach an endpoint than expected. Therefore, by using an endpoint of short duration (5 min), it may result in readings being taken before the reaction is complete, leading to lower TEAC values than the actual values [57].

The antioxidant properties of OFI extracts support their potential interaction with the redox‐sensitive transcription factors NF‐κB and NRF‐2. This interaction may contribute to the increased antioxidant capacity of OFI‐CE, which could explain the biological activity of the extract in fat cells.

Overall, our findings lend further support to the scientific understanding of the beneficial effects of utilizing OFI as a source for producing functional ingredients to combat oxidative stress and inflammation‐related diseases. To the best of our knowledge, there is limited data on the effects of OFI on the expression of inflammatory mediators in inflamed adipocytes or on the functional dynamics of adipocyte‐monocyte interactions. Our findings contribute to addressing this knowledge gap. Only one very recent study has clinically evaluated the effects of OFI supplementation in terms of modulating biomarkers of oxidative stress in healthy subjects, finding a significant increase in total salivary antioxidant capacity (*p* <0.001) and concomitant decreases in malondialdehyde, nitrotyrosine, and 8‐hydroxy‐2′‐deoxyguanosine (*p* <0.001) [58] thereby supporting the translational relevance of our data.

A limitation of our study is that the findings presented are based solely on human cell culture systems and cell‐free systems. Thus, while they provide insights into the mechanistic effects of OFI on metabolism, their applicability to patients is not assured, as only evidence from clinical trials can offer such certainty. Consequently, there is an evident need for further characterization and understanding of these properties to translate them into clinically safer nutraceuticals or dietary recommendations. However, our research deepens the mechanistic understanding of natural bioactives and highlights the potential of OFI as a versatile component in functional foods and nutraceutical formulations, with promising applications in the prevention of obesity‐related low‐grade inflammation.

## 5 Conclusion

In conclusion, according to the hypothesis, the extract of OFI cladodes demonstrates significant antioxidant and antiinflammatory effects in adipocytes. This finding highlights the potential of this plant species to be used as a dietary supplement or therapeutic agent. Its application in nutritional treatments could be beneficial not only for weight management but also for addressing the inflammatory markers associated with obesity. The dual functionality of OFI cladode polyphenols in reducing oxidative stress and inflammation makes them a promising candidate for integrative approaches to obesity treatment, enhancing overall metabolic health and mitigating obesity‐related complications.

## Conflicts of Interest

The authors declare no conflicts of interest.

## Peer Review

The peer review history for this article is available at https://publons.com/publon/10.1002/mnfr.70114.

## Data Availability

The authors confirm that the data supporting the findings of this study are available within the article.
